# Distinguishing Functional Amino Acid Covariation from Background Linkage Disequilibrium in HIV Protease and Reverse Transcriptase

**DOI:** 10.1371/journal.pone.0000814

**Published:** 2007-08-29

**Authors:** Qi Wang, Christopher Lee

**Affiliations:** 1 Center for Computational Biology, Molecular Biology Institute, Institute for Genomics and Proteomics, University of California at Los Angeles, Los Angeles, United States of America; 2 Department of Chemistry and Biochemistry, University of California at Los Angeles, Los Angeles, United States of America; Institute of Human Virology, United States of America

## Abstract

Correlated amino acid mutation analysis has been widely used to infer functional interactions between different sites in a protein. However, this analysis can be confounded by important phylogenetic effects broadly classifiable as background linkage disequilibrium (BLD). We have systematically separated the covariation induced by selective interactions between amino acids from background LD, using synonymous (S) *vs.* amino acid (A) mutations. Covariation between two amino acid mutations, (A,A), can be affected by selective interactions between amino acids, whereas covariation within (A,S) pairs or (S,S) pairs cannot. Our analysis of the *pol* gene — including the protease and the reverse transcriptase genes — in HIV reveals that (A,A) covariation levels are enormously higher than for either (A,S) or (S,S), and thus cannot be attributed to phylogenetic effects. The magnitude of these effects suggests that a large portion of (A,A) covariation in the HIV *pol* gene results from selective interactions. Inspection of the most prominent (A,A) interactions in the HIV *pol* gene showed that they are known sites of independently identified drug resistance mutations, and physically cluster around the drug binding site. Moreover, the specific set of (A,A) interaction pairs was reproducible in different drug treatment studies, and vanished in untreated HIV samples. The (S,S) covariation curves measured a low but detectable level of background LD in HIV.

## Introduction

Correlated amino acid mutation analysis has been widely used to infer functional interactions between different sites in a protein [1]–[12]. Typically, a strong correlation between amino acid mutations is interpreted as evidence of functional interactions under substantial selection pressure. For example, statistical covariation of amino acid mutations in HIV has revealed interesting biological interactions between sites, and constraints imposed by protein structure [13]–[19]. Therefore, studying covariation of amino acid mutations in HIV will improve our understanding of HIV drug resistance as well as help vaccine design [13], [18]. Studies of covariation in different regions of HIV genome have identified a number of correlated amino acid mutation pairs, many of which have known biological interactions [13]–[19].

**Figure 1:**
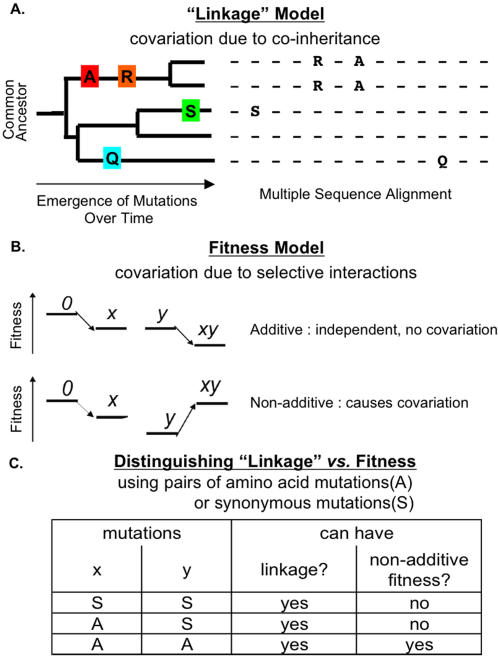
Schema of Separating Selective Interactions from Background Linkage Disequilibrium (BLD). (A) Mutation covariation due to BLD. Covariation of mutation A and R (shown in multiple sequence alignment, right) is caused by co-inheritance of the two mutations from a common ancestor (shown in the phylogenetic tree, left). (B) Mutation covariation due to selective interactions. Relative fitness models for mutations x and y, the double mutant (xy), and wildtype (0). Two models are contrasted: top, independent (additive) fitness effects don't cause amino acid mutation covariation; bottom, selective interactions cause covariation of x and y. (C) Distinguishing BLD *vs.* fitness using pairs of amino acid mutations (A) and synonymous (S) mutations.

However, such covariation analysis can be confounded by important phylogenetic effects [13], [14]. One major challenge for covariation analysis is distinguishing covariation that is genuinely due to selection pressure, from covariation that is simply due to co-inheritance from a common ancestor. When a mutation first occurs in an individual chromosome, other mutations are already present in that chromosome, and initially this mutation will be inherited in 100% linkage with those other mutations. Such co-occurrence due to common ancestry is classified as background linkage disequilibrium (BLD) [20] (Fig. 1A). Over time, however, such linkage will be scrambled by events such as recombination and mutation, returning to equilibrium (no statistical association between them). For example, homologous recombination events between any pair of mutations will gradually scramble any linkage between the mutation pair at a rate that is proportional to the physical distance between them, the recombination rate, and the passage of time. This raises several questions. How to distinguish BLD from the covariation due to selection pressure? What fraction of covariation is BLD? How strong is BLD in HIV? The evidence from different studies has been ambiguous. On one hand, studies indicate that phylogenetic effects in HIV are strong. Phylogenetic analysis has successfully inferred HIV transmission history from HIV sequences [21], [22]. On the other hand, HIV's high mutation rate [23], [24], recombination rate [25]–[27], and short generation time [28]–[30] should reduce the phylogenetic effect significantly.

It should also be emphasized that phylogenetic analysis can be confounded by strong selection pressure. Whereas phylogenetic analysis interprets the presence of the same mutation in several individuals as evidence of common ancestry, selection pressure creates bias for “convergent evolution” [31] in which the same mutation can evolve independently many times due to positive selection. Such hidden biases are incompatible with the assumptions of classical phylogenetic analysis [32]. For example, one study has reported that selection pressure for drug resistance can cause incorrect phylogenetic inferences from HIV sequences (compared with the known transmission history) [33].

Thus, it is important to develop methods that can distinguish these two causes of covariation. Recently, analytical methods such as parametric bootstrap [34] and phylogeny-based shuffling [35] have been applied to estimate what fraction of covariation arises from phylogenetic effects. In this paper we take a different approach, based on comparing levels of covariation between different types of mutation pairs, such as pairs of amino acid mutations (A,A) *vs.* pairs of synonymous mutations (S,S). Whereas (A,A) pairs are subject to both phylogenetic effects and selection pressure on functional interactions between amino acid sites, (S,S) pairs cannot be subject to this type of selection pressure (since they leave the amino acid sequence unchanged) (Fig. 1). We have therefore used synonymous mutations to measure background LD [20], to systematically distinguish covariation from phylogenetic effects *vs.* other sources of covariation, independent of mathematical model assumptions. Throughout this paper we will use the term “covariation” to refer to observed statistical association (without implying any specific interpretation of its cause); “background linkage disequilibrium” to indicate the specific interpretation of co-inheritance from a common ancestor; and “selective interactions” to indicate the specific interpretation of selection pressure for co-occurrence of a given pair of amino acid mutations. The “selective interactions” include 3D structural interactions (both local and long-range) as well as phenotypic covariation due to shared selection pressure. It should also be noted that selection on nucleotides (*e.g.* constraints on the RNA structure in viruses [36], [37]) rather than on amino acids can also cause BLD, as measured by (S,S) covariation, thus may contribute to the covariation of all three types of mutation pairs, (A,A), (A,S) and (S,S), in this study.

## Results

### Metrics of LD in HIV and Its Comparison with Background LD

First, we performed standard analyses of Linkage Disequilibrium (LD) on a dataset of about 50,000 HIV-1 *pol* gene sequences of subtype B, covering a 1.4 kb region of the HIV protease and reverse transcriptase (RT) genes, mostly from patients under antiretroviral drug treatment [38]. Following the procedure of the Human Genome HapMap project [39], we applied a minimum frequency criteria to the data before measuring the LD. After applying the frequency cutoff of 2%, our dataset included 398 distinct single nucleotide mutations, each with 3260 observation counts on average. It should be noted that due to the very large size of this dataset and the high rate of mutation in HIV, we detected a very high density of mutations, including mutations at the majority of individual nucleotide sites, most with large numbers of observations. This provided a uniquely high-resolution mutation dataset for mapping LD. The density of mutations (observations per nucleotide) in this dataset is 100-fold higher than in the data from the Human Genome HapMap project [39].

**Figure 2:**
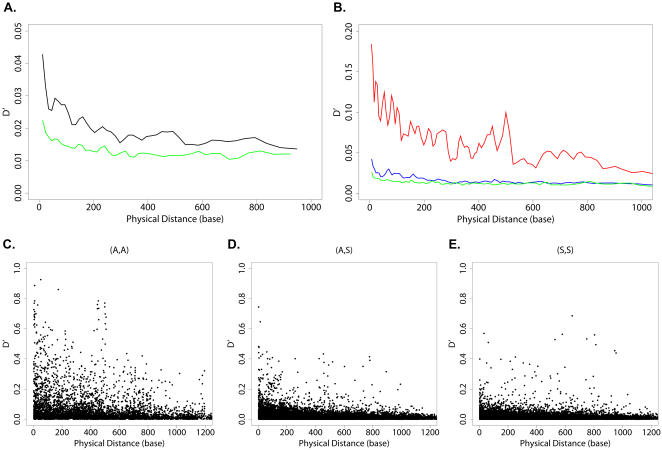
(A,A) Covariation Is Dramatically Higher Than (A,S) and (S,S) Covariation in the Specialty Dataset. (A) Sliding window results of average D′. All mutation pairs, black; silent mutation pairs (S,S) only, green. Each sliding window contains 4% of the data points in the set. (B) Sliding window results of average D′. Amino acid mutation pairs (A,A), red; amino acid mutations to silent mutations (A,S), blue; silent mutation pairs (S,S), green. Each sliding window contains 2% of the data points in the set. (C–E) Plots of D′ against the physical distance (base) within the mutation pair for C) (A,A), D) (A,S) and E) (S,S).

We computed D′ and r [40], two measures of statistical association commonly used to measure LD in many organisms, *e.g.* human [41]–[44]. Both metrics displayed a pattern in HIV similar to that in human, decaying as a function of distance (Fig. 2A, and Fig. S1A), as expected from population genetics theory. However, they indicated weaker LD than that in human [41]–[44], which is consistent with HIV's high mutation rate [23], [24], recombination rate [25]–[27], and short generation time [28]–[30], the factors that reduce LD according to the population genetics theory.

Another crucial difference between HIV and classical examples of LD analysis (*i.e.* the human data), is that this region of the HIV genome experiences very strong positive selection pressure due to antiviral drug treatments [45], [46]. To control for possible LD caused by amino acid selection pressure, we repeated this LD analysis strictly for pairs of synonymous mutations. Such mutations do not change the amino acid sequence and thus are not subject to amino acid selection pressure, yielding a measurement of “background LD” free of amino acid selection artifacts [20]. D′ and r metrics of the background LD decayed as a function of physical distance (Fig. 2A, and Fig. S1A). However, the average background LD by these metrics was smaller than the average LD, across the whole one kb region. The average D′ for background LD decayed from 0.02 for adjacent mutations to 0.01 for distances of 0.4 kb or more, about two-fold lower than the standard LD curve over the same distance range (Fig. 2A). The average r showed a similar pattern (Fig. S1A). This suggests that selection pressure plays an important role in shaping LD in HIV.

### Comparing Covariation of (A,A), (A,S) and (S,S) in HIV

To examine this hypothesis further, we subdivided mutation pairs into three groups: pairs of synonymous mutations (S,S); pairs of amino acid mutations (A,A); and pairs consisting of one amino acid mutation and one synonymous mutation (A,S). (A,A) pairs experience both phylogenetic effects and possible selective interactions; that is, (A,A) pairs that together increase reproductive fitness may be selected for co-occurrence. By contrast, since synonymous mutations do not affect the amino acid sequence, both (S,S) and (A,S) pairs are not subject to amino acid selective interaction effects. Thus, the extent of selective interaction effects can be estimated by a systematic pattern of excess covariation specifically for (A,A) pairs relative to that for (S,S) and (A,S) pairs.

We first compared the (A,A), (A,S) and (S,S) covariation in the same dataset of 50,000 HIV-1 samples. After applying minimum frequency cutoff of 2%, 124 amino acid mutations and 274 silent mutations were included, yielding 7626 (A,A) pairs, 33976 (A,S) pairs, and 37401 (S,S) pairs. Compared with (A,S) and (S,S), (A,A) covariation was dramatically higher, in both D′ and r (Fig. 2, and Fig. S1). Only (A,A) pairs showed D′ greater than 0.8 and many more (A,A) pairs showed strong covariation (D′>0.5) than (A,S) and (S,S) pairs (Fig. 2C, 2D, 2E). Furthermore, the average covariation of (A,A) was much higher than that of (A,S) and (S,S) (Fig. 2B). The average D′ of (A,A) gradually declined from 0.18 to 0.03 over about 1000 bases, while the average D′ of (A,S) and (A,A) started at less than 0.05 and rapidly dropped to 0.01 at around 300 bases. On average, (A,A) covariation levels were two- to five-fold higher than those of (A,S) and (S,S) across this range of distances. The conclusion also held for the frequency cutoff of 1% and 4% (Fig. S2 and S3). In addition, the difference in distribution for covariation scores D′ of (A,A) *vs.* those of (A,S) and for (A,A) *vs.* (S,S) was statistically significant (both p-values less than 10^−16^, Wilcoxon rank sum test — see Materials and Methods). Thus, a predominant fraction of (A,A) covariation does not appear to be attributable to background LD as measured by (S,S) covariation.

It is also striking that the (A,S) and (S,S) covariation (measured by D′ and r) behaved similarly, in contrast with (A,A) covariation. The average D′ of (A,S) and (S,S) both started under 0.05 and gradually decayed until they reached a flat of around 0.01 at 300 bases (Fig. 2B). The same pattern was repeated in the average r curve (Fig. S1B). However, it is also interesting that there appear to be slight differences between (A,S) and (S,S) at short distances (less than 200 bases). The average D′ value for (A,S) was significantly higher (up to 0.04) than (S,S) for adjacent mutations, but decayed more rapidly, so that this difference vanished beyond 300 bases. This higher value of (A,S) *vs.* (S,S) is consistent with the known strong positive selection for amino acid mutations in this region [45], [46], since (A,S) pairs would be directly affected by such potential selective sweep events [47], [48], whereas (S,S) pairs can only be affected indirectly (*i.e.* only by selective sweep for a third mutation that is a positively selected amino acid mutation).

### Comparing Covariation of (A,A), (A,S) and (S,S) in the Stanford-Treated Dataset

To assess the reproducibility of these results, we repeated this analysis of (A,A), (A,S) and (S,S) covariance in a second, independent dataset, containing about 7,000 drug-treated HIV samples of subtype B covering either protease or RT (Stanford-Treated; see Materials and Methods). 73 amino acid mutations and 103 silent mutations (mutation frequency ≥5%; see Materials and Methods) were included in the analysis.

**Figure 3:**
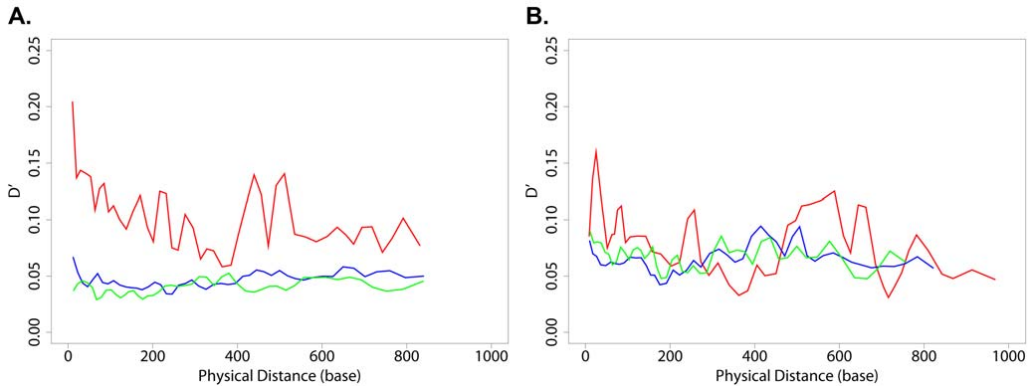
(A,A) Covariation Is Dramatically Higher than (A,S) and (S,S) Covariation in the Stanford-Treated Dataset but not the Stanford-Untreated Dataset. Sliding window results of average D′ in A) Stanford-Treated Dataset and B) Stanford-Untreated Dataset. Amino acid mutation pairs (A,A), red; amino acid mutations to silent mutations (A,S), blue; silent mutation pairs (S,S), green. Each sliding window contains 4% of the data points in the set.

Although the average number of samples per site in Stanford-Treated was less than one tenth of the Specialty dataset, we found the same covariance pattern — the (A,A) covariation (D′) was much stronger than that of (A,S) and (S,S) (both p-values less than 10^−7^, Wilcoxon rank sum test — see Materials and Methods), and the covariation levels of (A,S) and (S,S) were similar (p-value = 0.89, Wilcoxon rank sum test — see Materials and Methods). The average D′ of (A,A) started at around 0.20 and declined to 0.07 over a scale of 800 bases; while for (A,S) and (S,S), the average D′ started less than 0.07 and then both fluctuated at around 0.05 (Fig. 3A). The average r showed a similar pattern, differing from D′ mainly in measurement scale (Fig. S4A). Overall, (A,A) covariation was about two- to four-fold higher than (A,S) and (S,S) covariation levels. Again, the (A,S) and (S,S) curves were largely indistinguishable within the range of sampling variance inherent in the dataset (Fig. 3A). These data demonstrate again that most (A,A) covariation in this region of HIV is not attributable to background LD as measured by (S,S) covariation, suggesting a dominant role for selective interactions due to selection pressure imposed by antiviral drug treatment.

To exclude the possibility that the consistent pattern between Specialty and Stanford-Treated datasets results from overlapping samples, we eliminated from the Specialty dataset all sequences with 98% or higher identity to samples in the Stanford dataset and re-analyzed the Specialty dataset. After the filtering, the (A,A) covariation level is still much higher than (A,S) and (S,S) across all distances (Fig. S5).

### Comparing Covariation of (A,A), (A,S) and (S,S) in the Stanford-Untreated Dataset

To test the role of drug-induced selection via a negative control, we carried out the same analysis in a set of samples collected from untreated patients (Stanford-Untreated; see Materials and Methods). Previous studies have showed that comparison of these Treated *vs.* Untreated datasets can identify the effects of antiviral drug treatment [15], [18]. The Untreated dataset contained about 4,500 drug-naive samples covering either protease or RT (see Materials and Methods). 42 amino acid mutations and 107 silent mutations (mutation frequency ≥5%) were included in the analysis.

Strikingly, the large difference in covariation between (A,A) and (A,S)/(S,S) disappeared in the untreated dataset. The average D′ of (A,A) fluctuated around the average D′ of (A,S) and (S,S), at approximately 0.07 (Fig. 3B). The same pattern was repeated in the average r curve (Fig. S4B). These data provide a clear, independent confirmation that drug-induced selection pressure is indeed the explanation for the surplus covariation of (A,A) pairs (relative to background LD measured by (S,S)) in the Specialty and Stanford-Treated datasets, both of which included drug-treated samples.

### (A,A) Pairs Near the RT Active Site Show Strong Covariation

**Figure 4:**
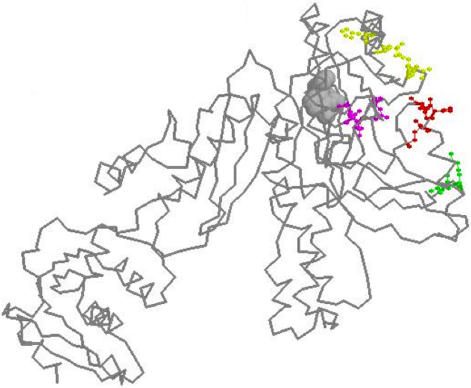
Amino Acid Mutation Pairs that Show Strong Covariation Are Close to Active Sites in RT. HIV-1 reverse transcriptase (RT) structure (PDB accession number 3HVTA) is shown using Protein Explorer (www.proteinexplorer.org). The RT41, 43 and 44, red; RT 67 and 70, green; RT 208, 210, 218, 219, yellow; active sites 110,185 and 186 in magenta. The grey sphere cluster is the nucleoside reverse transcriptase inhibitor — Nevirapine.

The (A,A) covariation decay curve (Fig. 2B) revealed a clear double-peak for pairs between 400–550 base distance in the Specialty dataset. Strikingly, a similar double-peak was observed at the same location in the Stanford-Treated (A,A) curve (Fig. 3A). We analyzed the two datasets separately to identify the mutation pairs responsible for these two peaks. These data revealed that the peaks were caused by the same set of mutation pairs in both datasets. One peak resulted from strong covariation between a cluster of mutations RT 41L and 43E with another cluster RT 208Y and 210W; while the other peak reflected strong covariation between a cluster RT 67N and 70R with the cluster RT 208Y, 218E and 219E/Q. Interestingly, in the three-dimensional protein structure, all these residues lie close to the reverse transcriptase active site (Fig. 4), less than 25 Å apart. Furthermore, mutations RT 41L, 67N, 70R, 210W and 219E/Q are known RT drug resistance mutation [49]. Thus every single one of the (A,A) covariation pairs observed in these peaks consisted of either one, or two known drug-resistance mutations. This analysis of the individual (A,A) covariation pairs provides independent confirmation that these specific residues are positively selected for drug-resistance.

### Comparison of Covariation Maps of Protease in the Specialty Dataset

**Figure 5:**
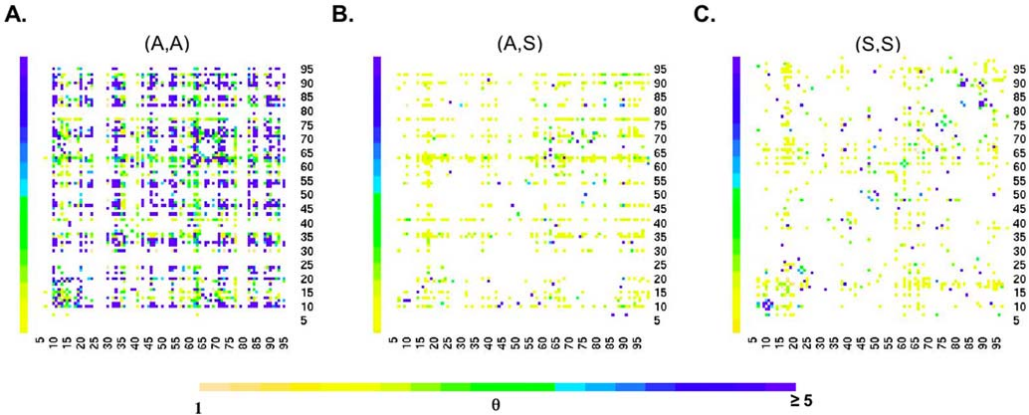
The Covariation Maps of Three Different Types of Mutation Pairs in HIV Protease. The covariation maps of A) amino acid mutation pairs (A,A), B) amino acid mutations to silent mutations (A,S) and C) silent mutation pairs (S,S). The X and Y axes represent the codon positions in protease. Each cell represents the strongest covariation value (θ; see Materials and Methods) measured for any mutation pair of the designated type between the two positions. The strength of the covariation is depicted on a color scale, with yellow indicating covariation score (θ) larger than 1 and varying shades up to blue indicating covariation score (θ) larger than 5 (the covariation of two mutations is at least five times greater than random). White indicates no evidence of covariation.

To provide a comprehensive analysis of the specific (A,A) pairs that showed significant evidence of selective interactions, we constructed two-dimensional maps of the statistical strength of covariation between all possible pairs of codons positions in HIV protease, for (A,A), (A,S) and (S,S) (Fig. 5). We used Fisher's exact test to calculate the covariation score θ (see Materials and Methods), which detects statistically significant covariation even in cases with smaller counts. No minimum frequency criteria was applied. This allows us to comprehensively compare the covariation of different types of mutation pairs. In this analysis, we used the Specialty dataset, due to its much larger number of samples (about 50,000). For each pair of codon positions, the map displays the highest level of covariation measured for mutations at that pair (see Materials and Methods).

These data reveal several striking differences between (A,A), (A,S) and (S,S) covariation. First, the (A,A) map contains a large fraction (2.6%) of strong covariation effects (θ>5 at 95% confidence; see Materials and Methods), compared with only a small fraction for (A,S) (0.3%) and (S,S) (0.3%). Second, whereas strong (A,A) covariation broadly distributed across the whole map (Fig. 5A), in the (A,S) and (S,S) maps covariation clustered close to the diagonal (Fig. 5B and 5C), *i.e.* for codon positions that are close in the sequence. These data suggest that background LD decays rapidly, within 100 nt (about 30 amino acids). The fact that (A,A) covariation extended more broadly, indicates that it arises from a different process than background LD. Third, a large fraction (30 out of 53) of the codon positions identified by (A,A) covariation are known drug-resistance mutation sites [49] in HIV protease (for the list of drug-resistance codons identified, see Materials and Methods), confirming again that these covariation effects involve drug-resistance selection.

### Correlation of the Covariation Between Independent Datasets

We have shown that the level of (A,A) covariation is higher than (A,S) and (S,S) covariation in both drug-treated datasets (Specialty; Stanford-Treated). However, do these independent datasets display the same covariation effects? To answer this question, we compared the covariation measurements for each (A,A) pair from these two datasets. For each (A,A) pair, we plotted the covariation value observed in the Specialty dataset against the covariation value observed in the Stanford-Treated dataset (Fig. S6). Strikingly, the (A,A) pairs that covaried in the Specialty dataset also covaried in the Stanford-Treated (Fig. S6A), and the covariation values in the two datasets showed strong quantitative agreement, yielding a high correlation coefficient of 0.83 (See Materials and Methods) between these two independent datasets. In contrast, for (A,S) and (S,S) pairs, the covariation detected in these two datasets was low, both giving the correlation coefficients of 0.35. This indicates that a single, consistent pattern of selective interactions is reproducibly discovered in the two independent datasets, but only for (A,A) covariation.

To assess whether drug treatment acts as the consistent amino acid selection in these two datasets, we compared the (A,A) covariation in the Treated dataset with that in the Untreated one. We found that the high consistence of (A,A) covariation between the Specialty and the Treated (correlation coefficient 0.83) disappeared in the comparison between the Untreated and the Treated, leaving a correlation coefficient of only 0.39 (Fig. S6D). This suggests that drug treatment (shared by the Specialty and the Treated datasets, but not the Untreated dataset) causes the nearly identical pattern of selective interactions found in these two independent datasets.

## Discussion

We have systematically separated the covariation induced by selective interactions from background LD, using silent (S) and amino acid (A) mutations. Selective interactions between amino acids can be detected by (A,A) pairs, but not by (A,S) or (S,S) pairs. Our analysis of the *pol* gene in HIV suggests that a large portion of (A,A) covariation in HIV results from selective interactions. Meanwhile, the (S,S) covariation curves suggest a low but detectable level of background LD in HIV. Although HIV has extremely high mutation and recombination rate, as well as short generation time, the (S,S) covariation metrics were still able to detect some BLD, decreasing as a function of physical distance (Fig. 2).

Several lines of evidence demonstrate the robustness of these conclusions. First, the same results were found by three different measurements of covariation: the widely used D′ and r metrics, and Fisher's exact test. Second, these results were reproduced in independent experimental studies (the Specialty and Stanford-Treated datasets). Third, the high level of consistency between independent (A,S) and (S,S) covariation curves suggests that the much higher level of covariation observed for (A,A) pairs cannot be attributed to background LD. Fourth, we also found direct evidence that the difference in covariation levels between (A,A) *vs.* (A,S)/(S,S) is due to selection, specifically, antiviral drug treatment, by comparing treated *vs.* untreated datasets. Fifth, the most prominent (A,A) interactions in the HIV *pol* gene have been independently identified as drug resistance mutations that physically cluster around the drug binding site. Finally, the specific set of (A,A) interaction pairs was reproducible in different drug treatment studies, and vanished in untreated HIV samples. Our result agrees with the ‘observation of positive epistasis in HIV [50]. A previous study in plastid genomes also indicates that the significant covariation in plastid genomes is likely due to changes in the selective constraints of amino acids [51].

Could the surplus of the (A,A) covariation compared with that of (A,S) and (S,S) in the treated datasets (Specialty and Stanford-Treated) be an artifact of differences in the intrinsic mutation rates between silent and amino acid mutations (*e.g.* silent mutations are more likely to be transitions than transversions, thus evolving faster)? We directly tested this possibility by performing the same analysis in samples from untreated patients (Stanford-Untreated). Such an artifact should have also have been observed in the untreated dataset. Yet, the difference between (A,A) *vs.* (A,S)/(S,S) disappeared in the untreated dataset (Fig. 3), indicating that this difference was due specifically to drug-treatment. It should also be noted that in addition to drug treatment, there are other sources of selection, such as immune pressure. Like the drug-induced selection, this too only causes (A,A) but not (A,S) or (S,S) covariation. However, we didn't detect a significant difference between (A,A) *vs.* (A,S)/(S,S) in the untreated samples, suggesting our approach is not sensitive enough to detect weaker selection.

How might drug treatment cause the dramatic increase in covariation of amino acid mutation pairs observed in HIV? Several models are possible. 1) Drug treatment selects for mutations that directly cause drug resistance (called primary mutations), many of which may have secondary effects such as reducing protein stability and/or other aspects of viral fitness. These mutations can in turn induce selection pressure for mutations that compensate for these effects (called accessory mutations; *e.g.* a mutation that restores the protein stability). Such secondary selection effects will cause a pattern of covariation of primary mutations with their associated accessory mutations. By contrast, in the untreated samples, where such positive selection forces are presumably weaker, we did not detect significant evidence of selective interactions. 2) The covariation can be caused by shared selection pressure among amino acid mutations. If mutation X and Y are independently selected for under the same drug treatment, the two mutations are likely to covary. To distinguish the aforementioned two possibilities, we need to estimate the fitness of mutation X and Y separately and compare the sum with the fitness of the XY double mutation, which is beyond the scope of this study.

Our data also indicate that accurate measurement of background LD is useful to improve the accuracy of functional interaction prediction. Accurate measurement of the background LD would enable calculation of a threshold value above which the statistics will have a specific probability of resulting from causes other than background LD. Comparison of statistical values of covariation calculated from (A,A) pairs with the background (*i.e.* (S,S) covariation) allows identification of pairs of sites having a specific probability of interacting due to selection on amino acids. Such phylogenetic effects should be taken into consideration in covariation analysis of amino acid interactions.

In this paper, we have only analyzed the *pol* gene, which is known to have experienced strong drug selection. The same method should be applied in the other regions of the HIV genome. We expect the (A,S) and (S,S) covariation, while still consistent with each other, varies across the HIV genome due to different phylogeny across the genome. The comparison of (A,A) covariation with that of (A,S)/(S,S) across the HIV genome will provide a global view of the influence of selection on mutation covariation. For example, such comparison in the *env* gene will hopefully improve our understanding of the interplay between the host selection and phylogeny in that region. The same analysis could also be done in subtypes other than subtype B.

## Materials and Methods

### HIV-1 sequence data

The Specialty dataset contained 48,927 subtype B sequences, mostly from patients under antiretroviral drug treatment. Multiple sequence alignments and mutation detection were performed as previously described [38], [52]. All these sequences covered the whole protease and part of the RT. The Treated and the Untreated datasets were downloaded from Stanford database (http://hivdb.stanford.edu/) [53], selecting only subtype B sequences. In the Treated dataset, there were 1795 protease sequences treated with protease inhibitor (this subset were used to calculate the covariation between mutations in protease) and 5121 reverse transcriptase sequences treated with either nucleoside reverse transcriptase inhibitor (NRI) or non-nucleoside reverse transcriptase inhibitor (NNRI) (this subset were used to calculate the covariation between mutations in RT), including 1320 samples that cover both protease and reverse transcriptase sequences (this subset were used to calculate the covariation between mutations in protease and those in RT). In each subset, we required a minimum mutation frequency of 5%. In the Untreated dataset, there were 2620 PI-naïve protease sequences and 1795 NRI/NNRI-naïve reverse transcriptase sequences, including 1208 samples that have both protease and reverse transcriptase treatment-naïve.

### Measurements of covariation for individual mutation pairs

We used the Fisher exact test [54], [55] to test for non-random associations between mutation α at position X and mutation β at position Y, by computing the p-value for the two-sided test using the 2×2 contingency table: N_XαYβ_, N_XαY0_, N_X0Yβ_ and N_X0Y0_·N_XαYβ_ is the number of samples that have mutation α at position X and also mutation β at position Y; N_XαY0_ is the number of samples that have mutation α at position X and but no kind of mutation at position Y; N_X0Yβ_ is the number of samples that have no mutation at position X and have mutation β at position Y; N_X0Y0_ is the number of samples that have mutation at neither position. We computed the odds ratio, its confidence interval (95% two-sided) and p-value using the fisher.test function from the statistical software package R. Note: the maximum likelihood estimator for θ is provided by (N_XαYβ_·N_X0Y0_)/(N_XαY0_·N_X0Yβ_); for independent mutations X_α_ and Y_β_, θ = 1. We calculated D′ and r following the standard procedures [56]–[58], using p_1_ = (N_XαYβ_+N_XαY0_)/N, q_1_ = (N_XαYβ_+N_X0Yβ_)/N, x_11_ = N_XαYβ_/N, where N = N_XαYβ_+N_XαY0_+N_X0Yβ_+N_X0Y0._. Finally, we used Wilcoxon rank sum test (wilcox.test function in the R package) to compare different types of mutation pairs with respect to their covariation score (*e.g.* D′). The p-value is calculated for the null hypothesis that the covariation scores for the two types of mutation pairs are from the same distribution.

### Average LD as a function of distance

Mutation pairs with negative LD were excluded. Mutation pairs were ranked by physical distance. We calculated smoothed curves using a sliding window, the window width of 2% or 4% of the total data, and an offset for neighboring windows of the 1/2 the window width.

### Covariation map

For each pair of mutations, we used Fisher exact test to compute a p-value for statistically significant covariation, along with a lower-bound estimate for the strength of covariation θ based on the 95% confidence interval. Only statistically significant mutation pairs (p<10^−6^ for a single pair, yielding a significance level of 0.01 after the Bonferroni correction) were included in our analysis. For a given codon position pair, the strongest covariation value θ for any pair of mutations at the two positions (of the designated type: (A,A), (A,S), or (S,S)) was displayed in the map.

In protease, the drug-resistant codons identified by (A,A) covariation are 10, 13, 16, 20, 24, 30, 32, 33, 35, 36, 43, 46, 47, 48, 50, 53, 54, 58, 60, 62, 63, 71, 73, 74, 82, 84, 85, 88, 90 and 93.

### Comparing covariation between datasets

To test whether the covariation derived from two datasets, X and Y, was consistent, we plotted for every mutation pair the covariation measurement r in X *vs.* that in Y. We also calculated the correlation coefficient between the two datasets. Since correlation coefficient is very sensitive to outliers, the lowest correlation from 2000 bootstrap replicates (the R boot library) was taken as the correlation score between X and Y.

## Supporting Information

Figure S1 (A,A) Covariation Measured by r Is Dramatically Higher than (A,S) and (S,S) Covariation in the Specialty Dataset. (A) Sliding window results of average r. All mutation pairs, black; silent mutation pairs (S,S) only, green. Each sliding window contains 4% of the data points in the set. (B) Sliding window results of average r. Amino acid mutation pairs (A,A), red; amino acid mutations to silent mutations (A,S), blue; silent mutation pairs (S,S), green. Each sliding window contains 2% of the data points in the set. (C–E) Plots of r against the physical distance (base) within the mutation pair for C) (A,A), D) (A,S) and E) (S,S).
(0.84 MB TIF)

Figure S2 (A,A) Covariation Is Still Dramatically Higher than (A,S) and (S,S) Covariation in the Specialty Dataset Using 0.01 As the Mutation Frequency Cutoff. (A–C) Plots of D' against the physical distance (base) within the mutation pair for A) amino acid mutation pairs (A,A), B) amino acid mutations to silent mutations (A,S) and C) silent mutation pairs (S,S). (D, E) Sliding window results of average D) D' and E) r. (A,A), red; (A,S), blue; (S,S), green. Each sliding window contains 2% of the data points in the set.
(0.88 MB TIF)

Figure S3 (A,A) Covariation Is Still Dramatically Higher than (A,S) and (S,S) Covariation in the Specialty Dataset Using 0.04 As the Mutation Frequency Cutoff. (A–C) Plots of D' against the physical distance (base) within the mutation pair for A) amino acid mutation pairs (A,A), B) amino acid mutations to silent mutations (A,S) and C) silent mutation pairs (S,S). (D, E) Sliding window results of average D) D' and E) r. (A,A), red; (A,S), blue; (S,S), green. Each sliding window contains 2% of the data points in the set.
(0.55 MB TIF)

Figure S4 (A,A) Covariation Measured by r Is Dramatically Higher than (A,S) and (S,S) Covariation in the Stanford-Treated Dataset But Not the Stanford-Untreated Dataset. Sliding window results of average r in (A) Stanford-Treated Dataset and (B) Stanford-Untreated Dataset. Amino acid mutation pairs (A,A), red; amino acid mutations to silent mutations (A,S), blue; silent mutation pairs (S,S), green. Each sliding window contains 4% of the data points in the set.
(0.17 MB TIF)

Figure S5 (A,A) Covariation Is Still Dramatically Higher than (A,S) and (S,S) Covariation in the Specialty Dataset After Excluding Samples That Have Nucleotide Sequence Similarity 98% Or Greater With Any Sample In the Stanford-Treated Dataset. (A–C) Plots of D' against the physical distance (base) within the mutation pair for A) amino acid mutation pairs (A,A), B) amino acid mutations to silent mutations (A,S) and C) silent mutation pairs (S,S). (D, E) Sliding window results of average D) D' and E) r. (A,A), red; (A,S), blue; (S,S), green. Each sliding window contains 2% of the data points in the set.
(0.88 MB TIF)

Figure S6 Shared Drug Treatment Leads to High Consistency of Amino Acid Covariation between Independent Datasets. (A–C) The covariation measurement r in the Specialty dataset plotted against that in the Stanford-Treated dataset for A) amino acid mutation pairs (A,A), B) amino acid mutations to silent mutations (A,S) and C) silent mutation pairs (S,S). (D–F) The covariation measurement r in the Stanford-Untreated dataset plotted against that in the Treated dataset, for D) (A,A), E) (A,S) and F) (S,S).
(0.65 MB TIF)

## References

[1] Altschuh D, Lesk AM, Bloomer AC, Klug A (1987). Correlation of co-ordinated amino acid substitutions with function in viruses related to tobacco mosaic virus.. J Mol Biol.

[2] Gobel U, Sander C, Schneider R, Valencia A (1994). Correlated mutations and residue contacts in proteins.. Proteins.

[3] Shindyalov IN, Kolchanov NA, Sander C (1994). Can three-dimensional contacts in protein structures be predicted by analysis of correlated mutations?. Protein Eng.

[4] Thomas DJ, Casari G, Sander C (1996). The prediction of protein contacts from multiple sequence alignments.. Protein Eng.

[5] Olmea O, Valencia A (1997). Improving contact predictions by the combination of correlated mutations and other sources of sequence information.. Fold Des.

[6] Olmea O, Rost B, Valencia A (1999). Effective use of sequence correlation and conservation in fold recognition.. J Mol Biol.

[7] Larson SM, Di Nardo AA, Davidson AR (2000). Analysis of covariation in an SH3 domain sequence alignment: applications in tertiary contact prediction and the design of compensating hydrophobic core substitutions.. J Mol Biol.

[8] Fariselli P, Olmea O, Valencia A, Casadio R (2001). Progress in predicting inter-residue contacts of proteins with neural networks and correlated mutations.. Proteins Suppl.

[9] Fodor AA, Aldrich RW (2004). Influence of conservation on calculations of amino acid covariance in multiple sequence alignments.. Proteins.

[10] Suel GM, Lockless SW, Wall MA, Ranganathan R (2003). Evolutionarily conserved networks of residues mediate allosteric communication in proteins.. Nat Struct Biol.

[11] Lockless SW, Ranganathan R (1999). Evolutionarily conserved pathways of energetic connectivity in protein families.. Science.

[12] Hatley ME, Lockless SW, Gibson SK, Gilman AG, Ranganathan R (2003). Allosteric determinants in guanine nucleotide-binding proteins.. Proc Natl Acad Sci U S A.

[13] Korber BT, Farber RM, Wolpert DH, Lapedes AS (1993). Covariation of mutations in the V3 loop of human immunodeficiency virus type 1 envelope protein: an information theoretic analysis.. Proc Natl Acad Sci U S A.

[14] Bickel PJ, Cosman PC, Olshen RA, Spector PC, Rodrigo AG (1996). Covariability of V3 loop amino acids.. AIDS Res Hum Retroviruses.

[15] Gonzales MJ, Wu TD, Taylor J, Belitskaya I, Kantor R (2003). Extended spectrum of HIV-1 reverse transcriptase mutations in patients receiving multiple nucleoside analog inhibitors.. Aids.

[16] Gilbert PB, Novitsky V, Essex M (2005). Covariability of selected amino acid positions for HIV type 1 subtypes C and B.. AIDS Res Hum Retroviruses.

[17] Hoffman NG, Schiffer CA, Swanstrom R (2003). Covariation of amino acid positions in HIV-1 protease.. Virology.

[18] Wu TD, Schiffer CA, Gonzales MJ, Taylor J, Kantor R (2003). Mutation patterns and structural correlates in human immunodeficiency virus type 1 protease following different protease inhibitor treatments.. J Virol.

[19] Svicher V, Ceccherini-Silberstein F, Erba F, Santoro M, Gori C (2005). Novel human immunodeficiency virus type 1 protease mutations potentially involved in resistance to protease inhibitors.. Antimicrob Agents Chemother.

[20] Service SK, Ophoff RA, Freimer NB (2001). The genome-wide distribution of background linkage disequilibrium in a population isolate.. Hum Mol Genet.

[21] Leitner T, Escanilla D, Franzen C, Uhlen M, Albert J (1996). Accurate reconstruction of a known HIV-1 transmission history by phylogenetic tree analysis.. Proc Natl Acad Sci U S A.

[22] Thomson MM, Najera R (2005). Molecular epidemiology of HIV-1 variants in the global AIDS pandemic: an update.. AIDS Rev.

[23] Mansky LM, Temin HM (1995). Lower in vivo mutation rate of human immunodeficiency virus type 1 than that predicted from the fidelity of purified reverse transcriptase.. J Virol.

[24] Korber B, Theiler J, Wolinsky S (1998). Limitations of a molecular clock applied to considerations of the origin of HIV-1.. Science.

[25] Jetzt AE, Yu H, Klarmann GJ, Ron Y, Preston BD (2000). High rate of recombination throughout the human immunodeficiency virus type 1 genome.. J Virol.

[26] Zhuang J, Jetzt AE, Sun G, Yu H, Klarmann G (2002). Human immunodeficiency virus type 1 recombination: rate, fidelity, and putative hot spots.. J Virol.

[27] Rhodes T, Wargo H, Hu WS (2003). High rates of human immunodeficiency virus type 1 recombination: near-random segregation of markers one kilobase apart in one round of viral replication.. J Virol.

[28] Wei X, Ghosh SK, Taylor ME, Johnson VA, Emini EA (1995). Viral dynamics in human immunodeficiency virus type 1 infection.. Nature.

[29] Ho DD, Neumann AU, Perelson AS, Chen W, Leonard JM (1995). Rapid turnover of plasma virions and CD4 lymphocytes in HIV-1 infection.. Nature.

[30] Perelson AS, Neumann AU, Markowitz M, Leonard JM, Ho DD (1996). HIV-1 dynamics in vivo: virion clearance rate, infected cell life-span, and viral generation time.. Science.

[31] Doolittle RF (1994). Convergent evolution: the need to be explicit.. Trends Biochem Sci.

[32] Felsenstein J (1978). Cases in Which Parsimony or Compatibility Methods Will Be Positively Misleading.. Systematic Zoology.

[33] Lemey P, Derdelinckx I, Rambaut A, Van Laethem K, Dumont S (2005). Molecular footprint of drug-selective pressure in a human immunodeficiency virus transmission chain.. J Virol.

[34] Wollenberg KR, Atchley WR (2000). Separation of phylogenetic and functional associations in biological sequences by using the parametric bootstrap.. Proc Natl Acad Sci U S A.

[35] Noivirt O, Eisenstein M, Horovitz A (2005). Detection and reduction of evolutionary noise in correlated mutation analysis.. Protein Eng Des Sel.

[36] Hofacker IL, Stadler PF (1999). Automatic detection of conserved base pairing patterns in RNA virus genomes.. Comput Chem.

[37] Shapiro B, Rambaut A, Pybus OG, Holmes EC (2006). A phylogenetic method for detecting positive epistasis in gene sequences and its application to RNA virus evolution.. Mol Biol Evol.

[38] Chen L, Perlina A, Lee CJ (2004). Positive selection detection in 40,000 human immunodeficiency virus (HIV) type 1 sequences automatically identifies drug resistance and positive fitness mutations in HIV protease and reverse transcriptase.. J Virol.

[39] The International HapMap Consortium (2005). A haplotype map of the human genome.. Nature.

[40] Balding DJ (2006). A tutorial on statistical methods for population association studies.. Nat Rev Genet.

[41] Pritchard JK, Przeworski M (2001). Linkage disequilibrium in humans: models and data.. Am J Hum Genet.

[42] Reich DE, Cargill M, Bolk S, Ireland J, Sabeti PC (2001). Linkage disequilibrium in the human genome.. Nature.

[43] Dawson E, Abecasis GR, Bumpstead S, Chen Y, Hunt S (2002). A first-generation linkage disequilibrium map of human chromosome 22.. Nature.

[44] Stephens JC, Schneider JA, Tanguay DA, Choi J, Acharya T (2001). Haplotype variation and linkage disequilibrium in 313 human genes.. Science.

[45] Shafer RW (2002). Genotypic testing for human immunodeficiency virus type 1 drug resistance.. Clin Microbiol Rev.

[46] Clavel F, Hance AJ (2004). HIV drug resistance.. N Engl J Med.

[47] Smith JM, Haigh J (1974). Hitch-Hiking Effect of a Favorable Gene.. Genetical Research.

[48] Nielsen R, Williamson S, Kim Y, Hubisz MJ, Clark AG (2005). Genomic scans for selective sweeps using SNP data.. Genome Research.

[49] Johnson VA, Brun-Vezinet F, Clotet B, Kuritzkes DR, Pillay D (2006). Update of the drug resistance mutations in HIV-1: Fall 2006.. Top HIV Med.

[50] Bonhoeffer S, Chappey C, Parkin NT, Whitcomb JM, Petropoulos CJ (2004). Evidence for positive epistasis in HIV-1.. Science.

[51] Ane C, Burleigh JG, McMahon MM, Sanderson MJ (2005). Covarion structure in plastid genome evolution: a new statistical test.. Mol Biol Evol.

[52] Pan C, Kim J, Chen L, Wang Q, Lee C (2007). The HIV positive selection mutation database.. Nucleic Acids Res.

[53] Rhee SY, Gonzales MJ, Kantor R, Betts BJ, Ravela J (2003). Human immunodeficiency virus reverse transcriptase and protease sequence database.. Nucleic Acids Res.

[54] Fisher RA (1922). On the interpretation of x(2) from contingency tables, and the calculation of P.. Journal of the Royal Statistical Society.

[55] Agresti A (1992). A survey of exact inference for contingency tables.. Statistical Science.

[56] Brown AHD (1975). Sample Sizes Required to Detect Linkage Disequilibrium between 2 or 3 Loci.. Theoretical Population Biology.

[57] Lewontin RC (1988). On Measures of Gametic Disequilibrium.. Genetics.

[58] Ohta T, Kimura M (1970). Development of Associative Overdominance through Linkage Disequilibrium in Finite Populations.. Genetical Research.

