# Supplementary figures and images for: Distinguishing Functional Amino Acid Covariation from Background Linkage Disequilibrium in HIV Protease and Reverse Transcriptase
Source: PLoS One. 2007 Aug 29;2(8):e814. doi: 10.1371/journal.pone.0000814 (PMC1950573; doi:10.1371/journal.pone.0000814)

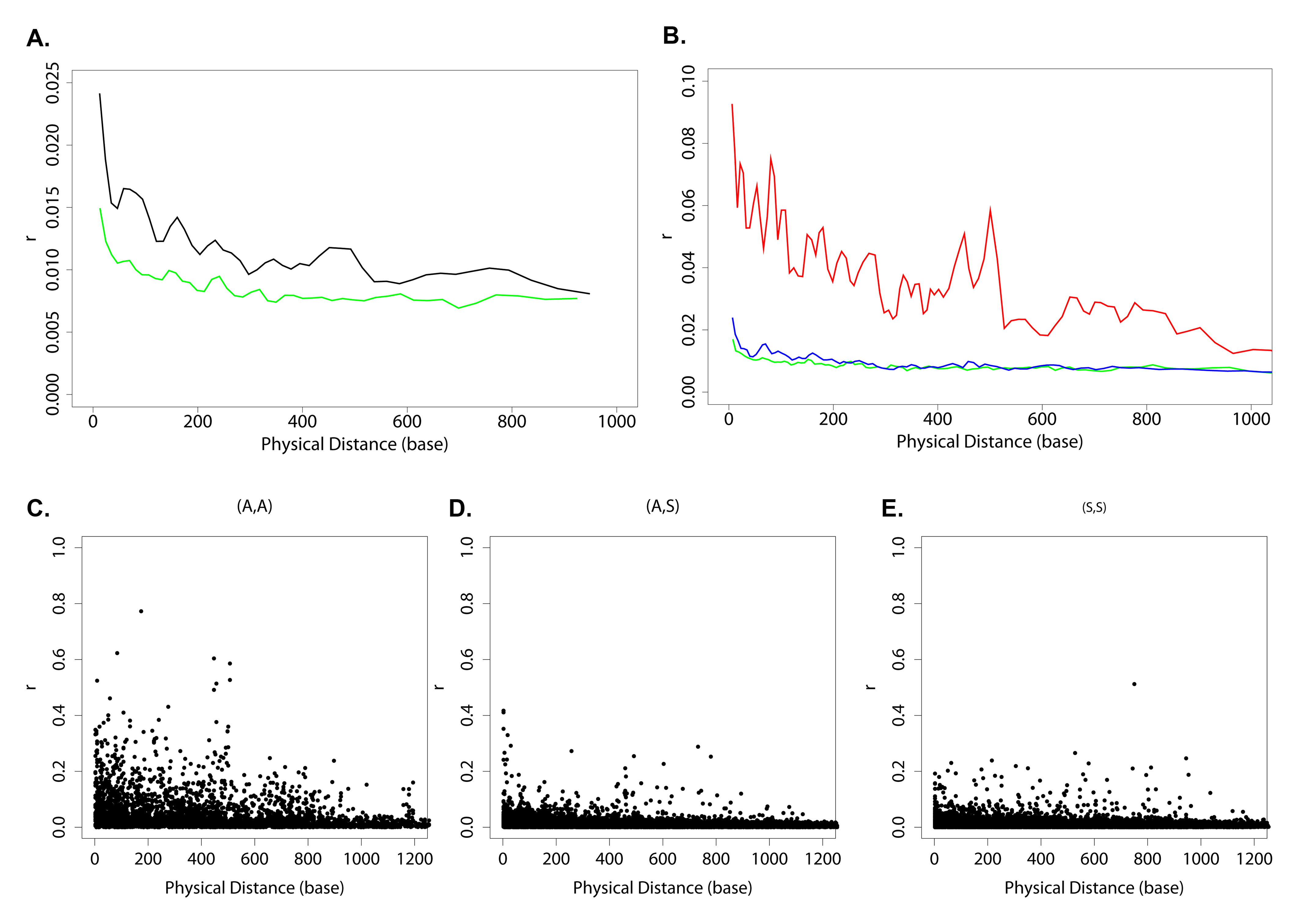

Supplement: Figure S1 — (A,A) Covariation Measured by r Is Dramatically Higher than (A,S) and (S,S) Covariation in the Specialty Dataset. (A) Sliding window results of average r. All mutation pairs, black; silent mutation pairs (S,S) only, green. Each sliding window contains 4% of the data points in the set. (B) Sliding window results of average r. Amino acid mutation pairs (A,A), red; amino acid mutations to silent mutations (A,S), blue; silent mutation pairs (S,S), green. Each sliding window contains 2% of the data points in the set. (C–E) Plots of r against the physical distance (base) within the mutation pair for C) (A,A), D) (A,S) and E) (S,S). (0.84 MB TIF) [file pone.0000814.s001.tif]

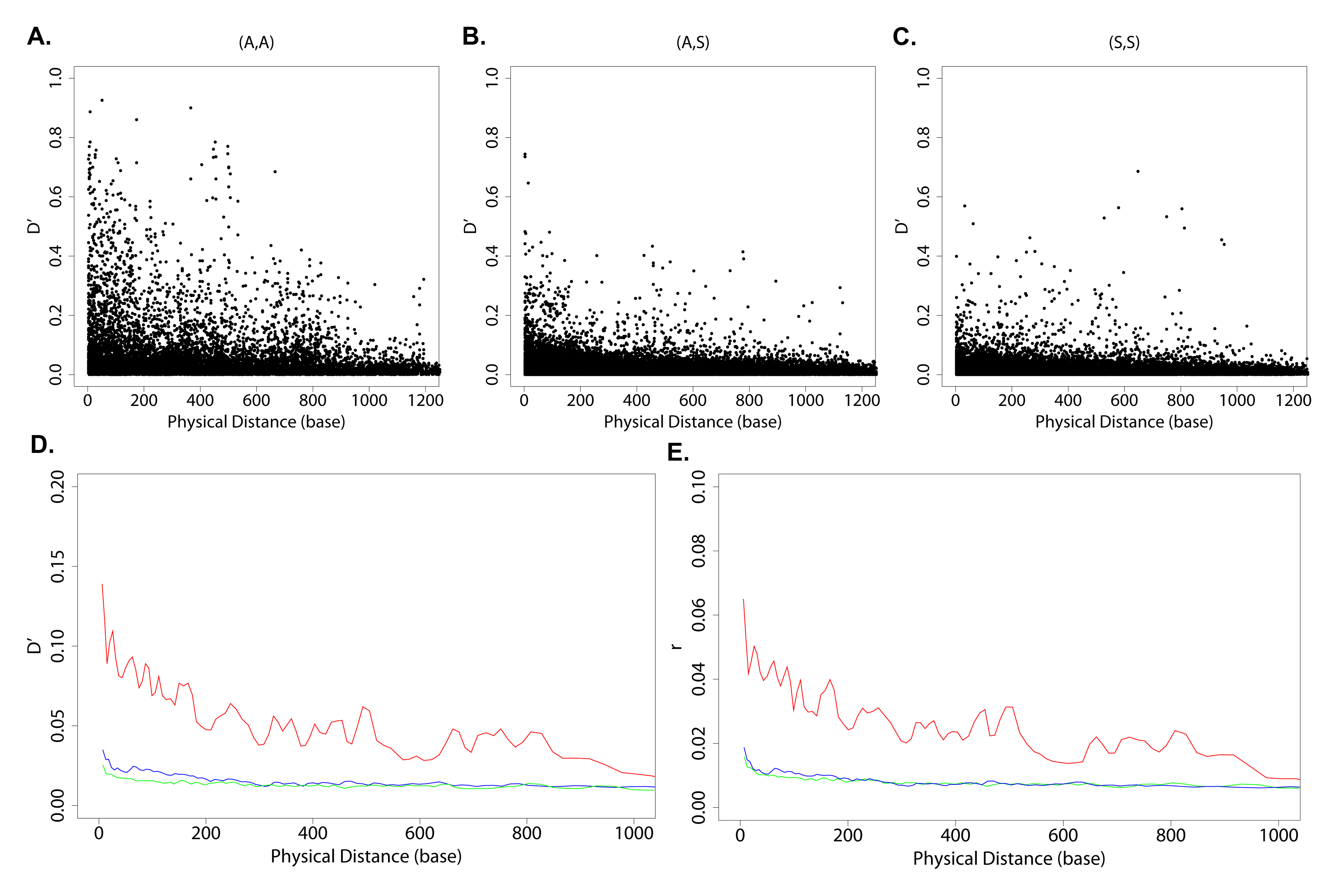

Supplement: Figure S2 — (A,A) Covariation Is Still Dramatically Higher than (A,S) and (S,S) Covariation in the Specialty Dataset Using 0.01 As the Mutation Frequency Cutoff. (A–C) Plots of D' against the physical distance (base) within the mutation pair for A) amino acid mutation pairs (A,A), B) amino acid mutations to silent mutations (A,S) and C) silent mutation pairs (S,S). (D, E) Sliding window results of average D) D' and E) r. (A,A), red; (A,S), blue; (S,S), green. Each sliding window contains 2% of the data points in the set. (0.88 MB TIF) [file pone.0000814.s002.tif]

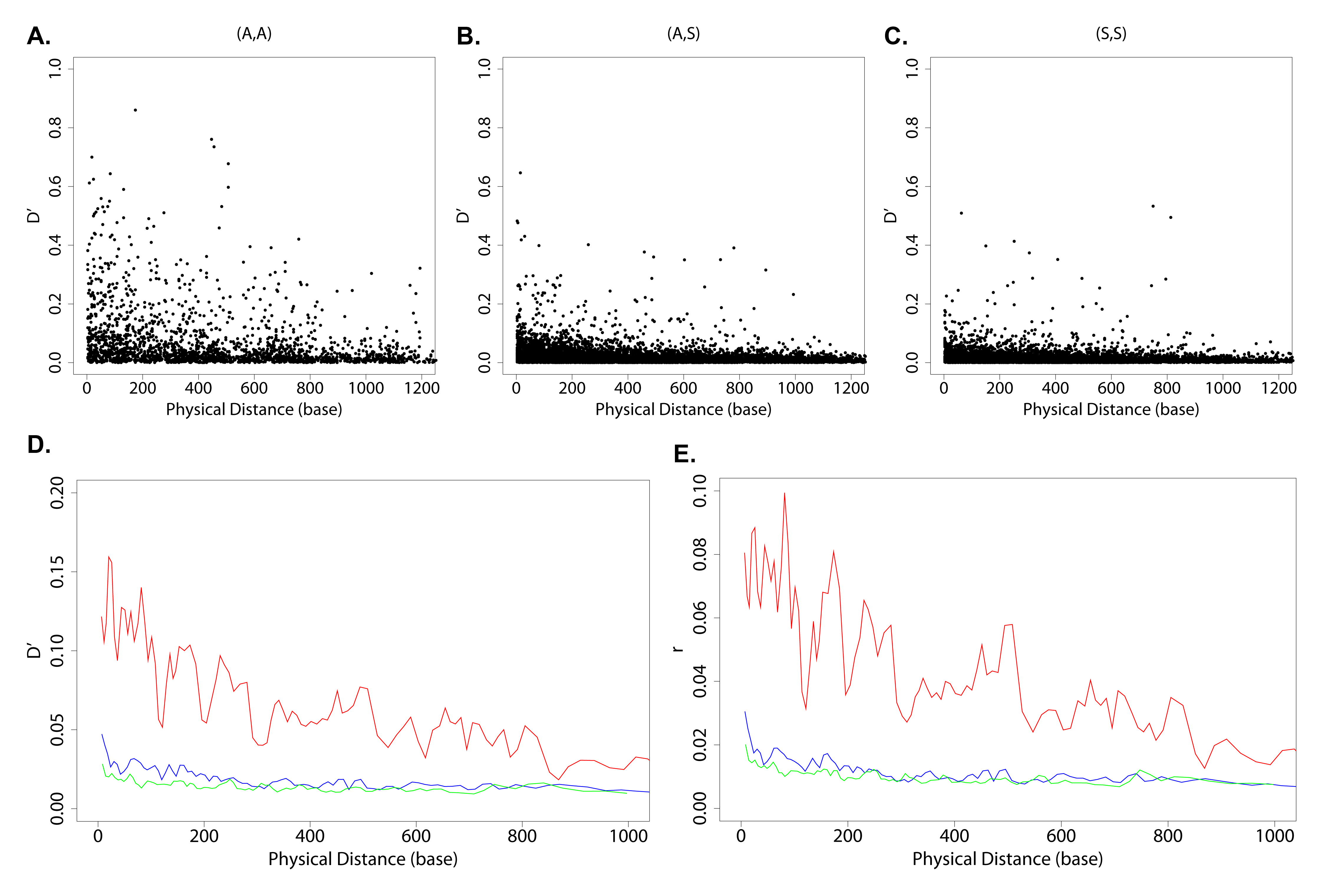

Supplement: Figure S3 — (A,A) Covariation Is Still Dramatically Higher than (A,S) and (S,S) Covariation in the Specialty Dataset Using 0.04 As the Mutation Frequency Cutoff. (A–C) Plots of D' against the physical distance (base) within the mutation pair for A) amino acid mutation pairs (A,A), B) amino acid mutations to silent mutations (A,S) and C) silent mutation pairs (S,S). (D, E) Sliding window results of average D) D' and E) r. (A,A), red; (A,S), blue; (S,S), green. Each sliding window contains 2% of the data points in the set. (0.55 MB TIF) [file pone.0000814.s003.tif]

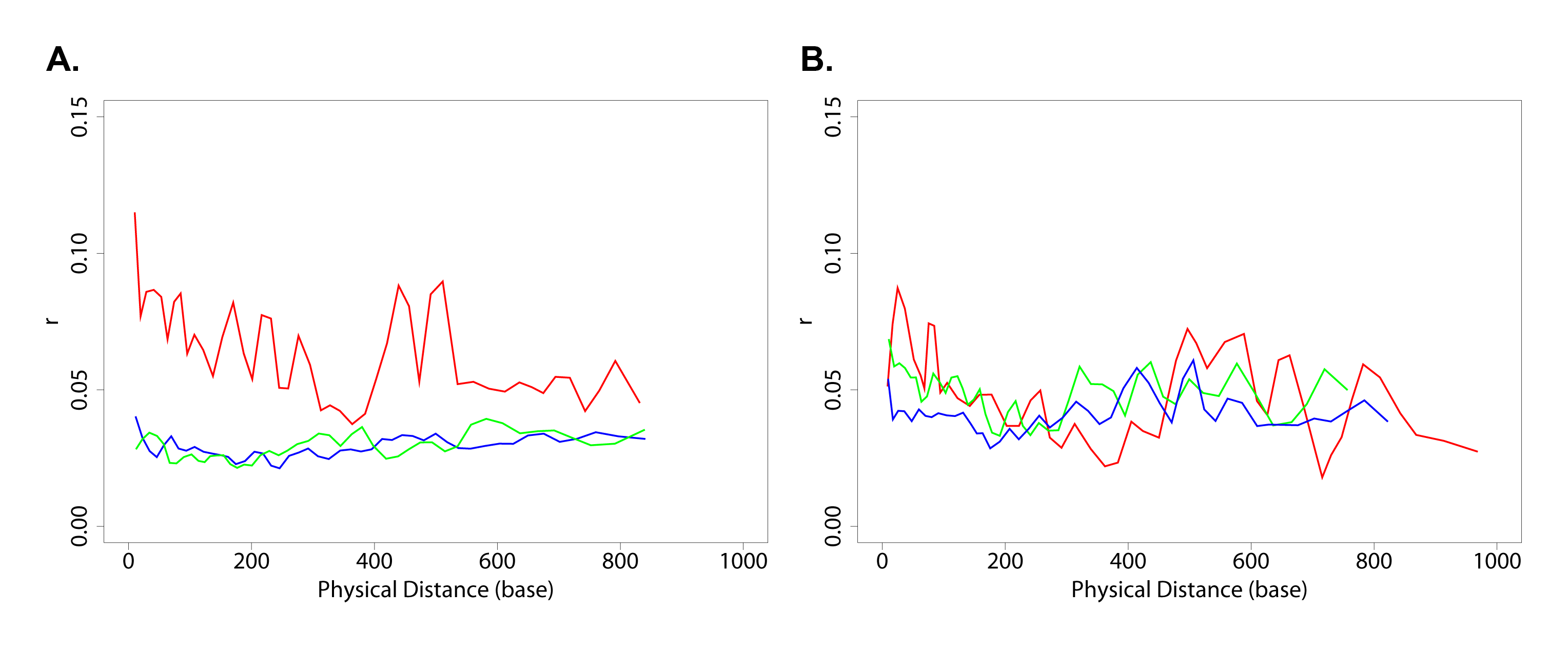

Supplement: Figure S4 — (A,A) Covariation Measured by r Is Dramatically Higher than (A,S) and (S,S) Covariation in the Stanford-Treated Dataset But Not the Stanford-Untreated Dataset. Sliding window results of average r in (A) Stanford-Treated Dataset and (B) Stanford-Untreated Dataset. Amino acid mutation pairs (A,A), red; amino acid mutations to silent mutations (A,S), blue; silent mutation pairs (S,S), green. Each sliding window contains 4% of the data points in the set. (0.17 MB TIF) [file pone.0000814.s004.tif]

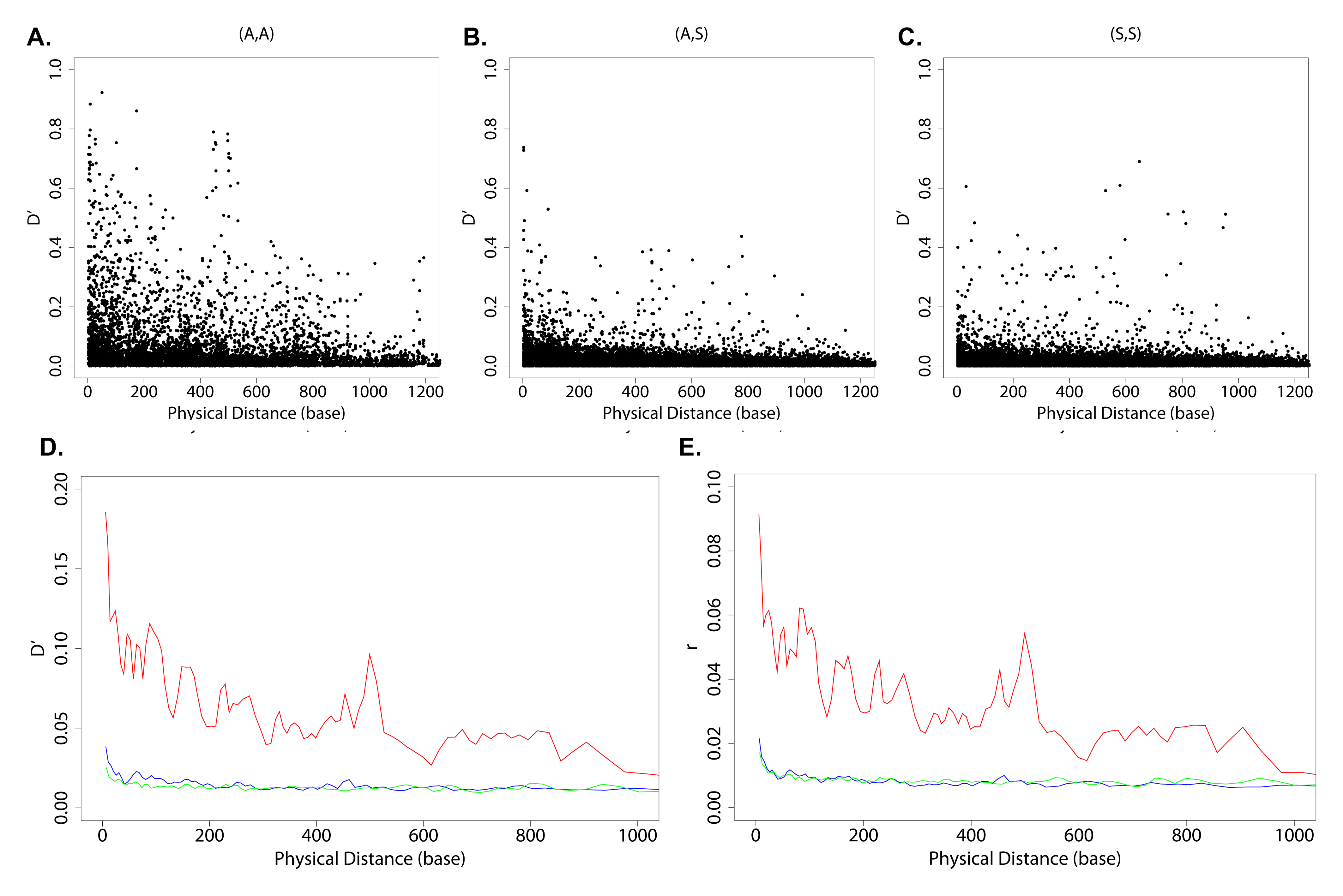

Supplement: Figure S5 — (A,A) Covariation Is Still Dramatically Higher than (A,S) and (S,S) Covariation in the Specialty Dataset After Excluding Samples That Have Nucleotide Sequence Similarity 98% Or Greater With Any Sample In the Stanford-Treated Dataset. (A–C) Plots of D' against the physical distance (base) within the mutation pair for A) amino acid mutation pairs (A,A), B) amino acid mutations to silent mutations (A,S) and C) silent mutation pairs (S,S). (D, E) Sliding window results of average D) D' and E) r. (A,A), red; (A,S), blue; (S,S), green. Each sliding window contains 2% of the data points in the set. (0.88 MB TIF) [file pone.0000814.s005.tif]

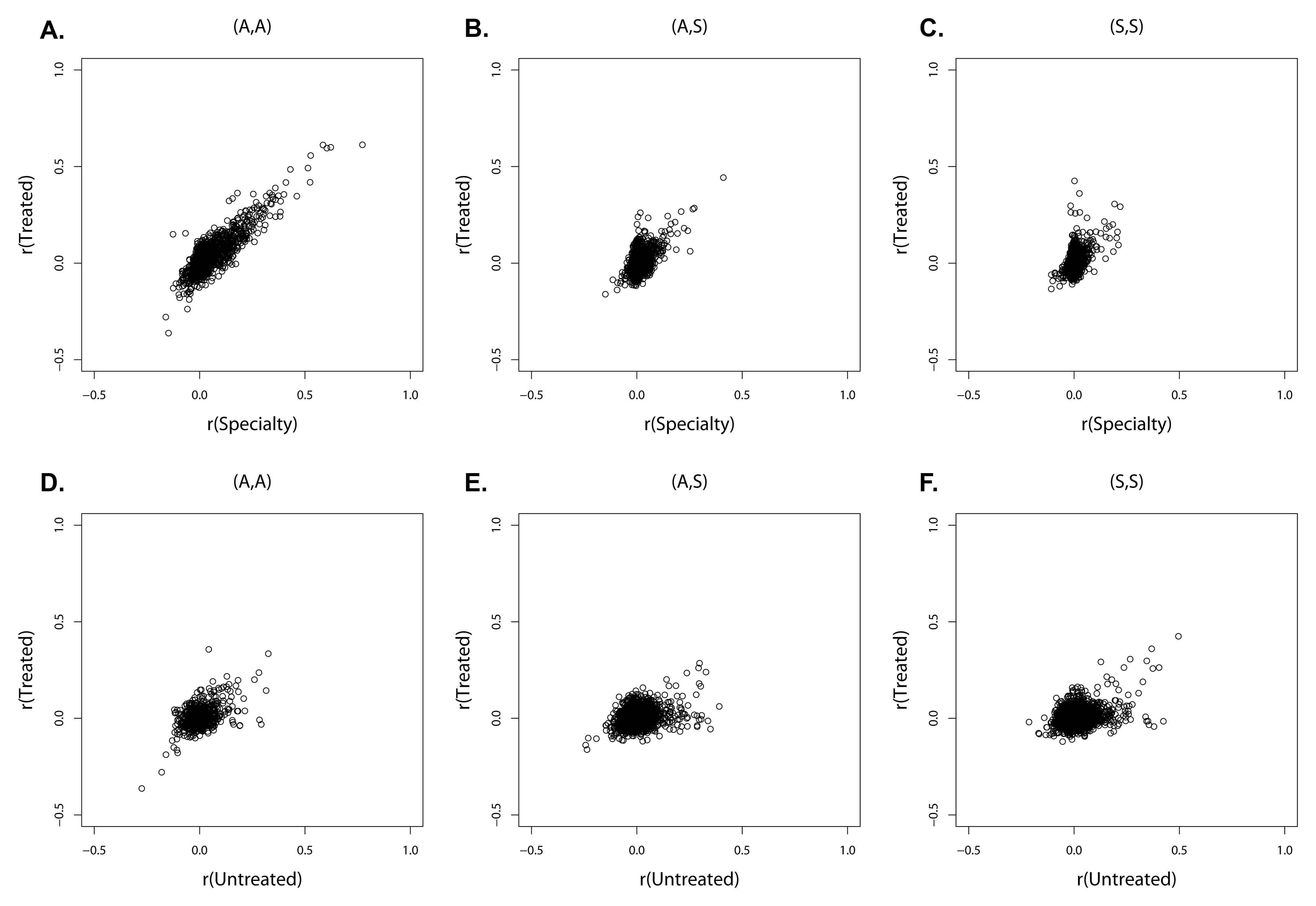

Supplement: Figure S6 — Shared Drug Treatment Leads to High Consistency of Amino Acid Covariation between Independent Datasets. (A–C) The covariation measurement r in the Specialty dataset plotted against that in the Stanford-Treated dataset for A) amino acid mutation pairs (A,A), B) amino acid mutations to silent mutations (A,S) and C) silent mutation pairs (S,S). (D–F) The covariation measurement r in the Stanford-Untreated dataset plotted against that in the Treated dataset, for D) (A,A), E) (A,S) and F) (S,S). (0.65 MB TIF) [file pone.0000814.s006.tif]
